# Life situation and support during pregnancy among Thai expectant mothers with depressive symptoms and their partners: a qualitative study

**DOI:** 10.1186/s12884-020-02914-y

**Published:** 2020-04-09

**Authors:** Nitikorn Phoosuwan, Pornpun Manasatchakun, Leif Eriksson, Pranee C. Lundberg

**Affiliations:** 1 Department of Public Health and Caring Sciences, Faculty of Medicine, Uppsala University, Uppsala, Sweden; 2 Department of Community Health, Faculty of Public Health, Kasetsart University Chalermphrakiat Sakonnakhon Province Campus, Sakon Nakhon, Thailand; 3 Boromarajonani College of Nursing Chiangmai, Chiangmai, Thailand

**Keywords:** Couples, Depressive symptoms, Expectant mother, Life situation, Support

## Abstract

**Background:**

Expectant parents may have positive and negative emotions during pregnancy and receive support from different sources. Studies on life situation and support among couples have rarely been conducted. This study aims to explore life situation and support during pregnancy among expectant mothers with depressive symptoms and their partners.

**Methods:**

Twenty-seven expectant mothers, in the last trimester of pregnancy with depressive symptoms (Edinburgh Postnatal Depression Scale score ≥ 7) from seven public antenatal care clinics in Sakonnakhon, a north-eastern province of Thailand, and their partners were interviewed. In total, 54 semi-structured interviews were subjected to content analysis.

**Results:**

Four categories emerged: (1) Having obstacles in life, (2) Facing life transition, (3) Enhancing confidence, and (4) Dissatisfaction with support. The informants described obstacles regarding economy, fear of health problems, getting an abnormal child and partners’ behaviours. They received support from family members and social networks, but some were dissatisfied with the support from the healthcare. For example, expectant fathers wished to receive more health information and be more involved.

**Conclusions:**

Healthcare professionals should be aware of the influence of cultural and contextual factors when providing antenatal care to expectant parents. Male involvement in the care must not be neglected.

## Background

Becoming a parent involves happiness and development from the confirmation of pregnancy until the first time holding the new-born child [1]. Throughout this period the parents try to adapt to the parental roles [2]. Depending on different factors and issues in their lives some expectant mothers (EMs) have positive emotions [1], while others have negative emotions [3]. Depression encompasses depressive symptoms (e.g. loss of interest and depressed mood) lasting at least 2 weeks [4]. It is one of the most common mental health problems during pregnancy, e.g. occurring among 12% of pregnant women in Italy and among 39% of pregnant women in Pakistan [1, 5]. In Thailand, the depression rate for women during late pregnancy is 10 times higher than that of non-pregnant women [6, 7].

Feelings of emotional isolation [3], financial strain [3, 6], history of depression, past childbearing experiences, low social support and lack of partner support [5] can cause antenatal depression, while having a strong family relationship contributes to good psychological health [8]. Depressive symptoms among EMs may cause depression in spouses, developmental problems for infants, and maternal suicide [5]. Thus, focusing on depressive symptoms among pregnant women is important.

Although only EMs are physically pregnant, expectant fathers (EFs) are equally important for their child [9]. They may have tension between different feelings about their future as fathers, and about 10% of them are depressed after childbirth [9, 10]. Fathers want to get involved and be informed from the beginning of pregnancy, but they are often ignored by healthcare professionals (HCPs) [9, 11]. Parental information provided at antenatal care (ANC) clinics during the early parenthood period is aimed at preparing couples for childbirth and parental roles [12]. During ANC it is equally important for HCPs to focus on the parents’ ability to cope with stress and on their knowledge and skills to care for the baby in order to strengthen them as individuals and as parents [3, 13].

In Thailand, pregnant women are offered ANC free of charge at ANC clinics in public hospitals or in contracted primary care centres where they are registered [13]. ANC is provided by nurses/midwives and includes maternal and child health screening of pregnancy-related diseases. Depression screening among EM is currently carried out with a non-specific tool with the two questions “During the past month, have you often been bothered by feeling down, depressed, distressed, or hopeless?” and “During the past month, have you often been bothered by little interest or pleasure in doing things?” [7], which may not accurately detect depressive symptoms. Screening tools such as the Edinburgh Postnatal Depression Scale (EPDS) [14] and diagnostic tools [15] may be more appropriate. The EPDS has been widely used both during pregnancy and after childbirth because it is simple to administer [1, 6, 14, 16]. Some studies focus on support needs among EMs and EFs [9, 11]. A systematic review [17] found that studies conducted about life situation during pregnancy among EMs with depressive symptoms and their partners were carried out mainly among those who were cohabiting and had a university level of education in western countries. As their quality of life decreased during pregnancy, domestic violence sometimes occurred. The results might differ from what Thai EMs and EFs experience because of the differences in context and culture (e.g. a mixture of Buddhists and Muslims, and a wide range of education) [5–7]. Additionally, there is a need for qualitative work to increase the understanding of the views of both EMs and EFs. Therefore, this study focused on expectant parents in the last trimester of pregnancy. The aim was to explore and describe life situation and support during pregnancy among EMs with depressive symptoms and their partners.

## Methods

### Study design

A descriptive and explorative design with individual semi-structured interviews [18] was employed in order to explore and describe life situation and support among EMs with depressive symptoms and their partners in the last trimester of pregnancy.

### Setting

This study was carried out in Sakonnakhon, a north-eastern province of Thailand. It is one of the ten provinces with the lowest income, has a mixture of religions (Buddhism, Christianity and Islam) and residential areas (i.e. rural and urban), and approximately 12,000 childbirths annually [6, 13]. Sakonnakhon has hospitals of three levels, namely provincial, general, and community hospitals, altogether eighteen hospitals. Seven hospitals (one provincial, one general and five community hospitals) were selected for data collection because they had a high number of annual childbirths for each level.

### Participants

Purposive sampling was used to select participants among EMs and their partners. The inclusion criteria for EMs were: (1) having received ANC in the selected hospitals in the last trimester of pregnancy (28–37 weeks gestational age), (2) having depressive symptoms (i.e. a score of seven or more from the Thai version of the EPDS tool), and (3) being willing to participate in this study. The exclusion criteria for EMs were: (1) did not live with their partners at time for data collection, and (2) reported a history of mental diseases. Based on previous use of the Thai version of the EPDS tool, a cut-off score of seven was considered suitable for detection of major and/or minor depression among Thai women [19]. The tool has been tested and used among pregnant women [6, 20]. EFs who were partners of the participating EMs were asked to participate voluntarily in individual interviews.

### Ethical approval

The study was approved by ethical review boards in Sweden (Dnr 2016/544) and in Thailand (SWDCPH2017–003) and was conducted in compliance with the ethical principles of the Declaration of Helsinki [20]. Permission to conduct this study was sought from the chief of Sakonnakhon Provincial Public Health Office. All participants received written and oral information before signing a written consent form. The information emphasised the freedom of partaking in the study or not and that participants were fully entitled to withdraw at any time. None of the authors was involved in the care of any of the participants.

### Data collection

Based on a literature review, three of the authors (NP, LE, and PCL) developed semi-structured interview guides for this study (one for EMs and one for EFs) about life situation and support during pregnancy for this study. The guides were tested on two couples in a province near Sakonnakhon and adjusted before use. Questions in the interview guides were open-ended in order to allow participants to describe their life situation and support during pregnancy. Initial questions about background information were followed by interview questions (see Additional file 1).

NP received a list of EMs in the last trimester of pregnancy from nurses/midwives at each hospital. Thereafter, by use of the EPDS, EMs were screened at their ANC clinics by NP and two research assistants who had been trained by NP in a previous study [6]. NP approached and asked EMs (with a wide range of ages, EPDS scores, religions and residential areas) with an EPDS score of seven or more and their partners (with a wide range of ages) to participate voluntarily in in-depth interviews. There was no screening of the participating EFs with the EPDS. Recruitment was stopped when nothing new emerged from the last interviews of EMs and their partners. Individual interviews were carried out in Thai during May and June 2017 by NP, PCL and PM, all Thai natives. NP had experience in public health and mental health. PM and PCL with experience from the field of nursing/midwifery, have PhD qualification and experience of qualitative research. In order to make participants feel comfortable [11], the female authors (PM and PCL) interviewed the EMs, while the male author (NP) interviewed the EFs. PM, PCL and NP took notes during the interviews. In addition, NP strongly advised the participating EMs to seek help from HCPs at their ANC clinic. The participants chose place (such as a room in a hospital or at home) and time for the interviews. Each EM and her partner were interviewed separately. At the end of each interview, the interviewer summarized the answers and the participants were able to clarify if there were any ambiguities. Each interview was recorded digitally and lasted about 60 min.

### Data analysis

Data for EMs and EFs were subjected to inductive qualitative content analysis [21]. All interviews were transcribed verbatim in Thai. Each Thai transcription was read and re-read several times by NP, PM and PCL who separately analysed the data. Data from EMs and their partners were first analysed separately as two data sets and then analysed together. All data from the interviews were divided into meaning units: words, statements and paragraphs, which reflect the principal meaning of the participants’ responses. The meaning units were condensed. Thereafter, codes with one word or a few words were created. Codes (*n* = 62) were compared and grouped into sub-categories and categories. Categories refer to a descriptive level of the content and an expression of the manifest content. Four interviews of two couples were translated from Thai to English and back. The English transcriptions, categories and sub-categories were rechecked by LE, a Swedish nurse with PhD-degree and experience in qualitative research. The final results were discussed, and categories and sub-categories were established. In addition, the final results were fed back to HCPs at district and provincial levels. The analytical process is presented in Table 1. Quotes in the result section are annotated with abbreviations (EM or EF) and a number. The number after ‘EPDS’ denotes the EPDS score. For example, EM7, EPDS10 refers to expectant mother number 7 with an EPDS score of 10.

**Table 1:** Examples of the analytical process

Meaning Units	Condensed meaning unit	Codes	Sub-categories	Categories
My problem is about money; I have to pay for the car and to take care of this child. I felt stressed about the money topic. (EM10)	Have problems with money and felt stress about expenses	Felt stress not enough money to use	Decreased dignity because of economic insufficiency	Having obstacles in life
I realise that I drank almost everyday. Now I’ve refrained for four days and during the three-month Buddhism period I intend to refrain from drinking. (EF12)	Try to refrain from drinking and plan to refrain longer	Refrain from drinking and don’t want to drink anymore	Need to prepare for a secure family	Facing life transition
After eight months, my mother-in-law will take care of the child in the afternoon when I go to work outside. (EM23)	Mother-in-law will take care of the child when the mother is working.	Assistance from mother-in-law	Getting encouragement from surrounding persons	Enhancing confidence
She (the nurse) saw the calendar and said that the calculation (for the expected date of childbirth) was wrong and she calculated it again. Then she went back and didn’t apologise to me.’ (EM8)	The staff made a mistake but did not apologise verbally.	Not enough verbal support from healthcare staff	Needing better healthcare services	Dissatisfaction with support

## Results

Twenty-seven couples participated in the study. Most EMs and EFs were 20 years old or more. About half of the EMs and most of the EFs were agriculturists or daily workers. The EPDS score of the EMs ranged from 7 to 23 out of 30. The participants’ demographic characteristics are presented in Table 2.

**Table 2:** Demographic characteristics of participants

Characteristics	Expectant mothers (*N* = 27)	Expectant fathers (*N* = 27)
Age, median (range)	29 (16–41)	29 (17–50)
< 20 years, n (%)	4 (14.8)	2 (7.4)
20 year or more, n (%)	23 (85.2)	25 (92.6)
Education, n (%)		
Secondary school or lower	14 (51.8)	17 (63.0)
High school or more	13 (48.2)	10 (37.0)
Occupation, n (%)		
No work	10 (37.0)	0
Agriculture/daily-work	13 (48.2)	24 (88.8)
Permanent work	4 (14.8)	3 (11.2)
Residential area^a^		
Rural	15 (55.6)	–
Urban	12 (44.4)	–
Religion^a^		
Buddhist	25 (92.6)	–
Christian	2 (7.4)	–
Gestational age, median (range)	35 (28–37)	–
Parity, median (range)	1 (0–4)	–
Multiparous, n (%)	17 (63.0)	–
Mothers’ EPDS score, median (range)	10 (7–23)	–
Marital status^a^, n (%)		
Married	20 (74.0)	
Live together	7 (26.0)	
Extended Family^a^, n (%)	19 (70.4)	
Year of living together^a^, Median (range)	3 (1–20)	
Less than 5 years, n (%)	16 (59.2)	
5 years or more, (n (%)	11 (40.7)	

^a^Data from the couples

Two categories of life situation in the last trimester of pregnancy were identified: (1) Having obstacles in life, and (2) Facing life transition. Two categories emerged about support during pregnancy: (1) Enhancing confidence, and (2) Dissatisfaction with support. Categories and sub-categories are presented in Table 3.

**Table 3:** Categories and sub-categories from the analysis

Categories	Sub-categories
Having obstacles in life	Decreased dignity because of economic insufficiency
	Fear of health problems, childbirth and an abnormal child
	Worry about partner’s behaviour
	Uncomfortable with in-law family members
Facing life transition	Need to prepare for a secure family
	Dealing with different life events
	Accepting changes in life
Enhancing confidence	Getting encouragement from surrounding persons
	Receiving assistance from social networks
Dissatisfaction with support	Needing better healthcare services
	Insufficient involvement by the family

### Having obstacles in life

The EMs and EFs felt that they were in a critical situation and had difficulties to manage their lives. Many of them experienced worry, fear, and stress in their current lives and could not adapt to the new situation. Therefore, they felt uncomfortable when interacting with their partners or in-law family members.

#### Decreased dignity because of economic insufficiency

All EMs and EFs explained that they were concerned about their economic situation. They mentioned that money was insufficient, and they needed to earn more money in order to increase the income of their family. EMs with EPDS score of 10 or more (17/27) often expressed this concern. Most EMs (23/27) had non-permanent work and did not work after getting pregnant. Hence, they had no income and used money from their partners who earned money. Most EFs (24/27) received pay per day and often did not have enough money for the needs of their family. The EMs (24/27) were worried about the economic situation for their family. In addition, some EFs (5/27) mentioned that having a child was an economic burden.

*I am worried about money. During pregnancy I have no work. I stay at home and I cannot help my partner to earn money. We will have some expenses because of the two-day admission in a hospital. My partner will take care of me but he cannot earn money.* (EM6, EPDS12)

*At the beginning, I was stunted. We are not ready to have a new child. We don’t have a long plan right now because we earn money on a daily basis. I plan to earn money instead of her (my wife) after childbirth.* (EF12)

#### Fear of health problems, childbirth and an abnormal child

The EMs and EFs were concerned about both mothers’ and children’s health because of problems found in the health check-up during pregnancy, for example, gestational diabetes. EMs (12/27), especially those who had bad previous experience from being pregnant (3/27), or who had heard rumours from other women (9/27), were afraid of experiencing pain during childbirth, having a difficult childbirth, having an infection or needing surgery. Some EMs (11/27) were also afraid that their child would be abnormal, weak or have a disease. Some EFs (7/27) were fearful due to information about the EMs’ complications of pregnancy.

*During pregnancy, I initially felt that it would be difficult to give birth and it would be painful. Last month, I had allergy symptoms…I also feared that I would not have enough force to give birth like before.* (EM16, EPDS10)

*Even though I am happy, I am worried about my wife’s haemorrhage.* (EF9)

#### Worry about partner’s behaviour

The EMs were worried about their partners’ behaviour. Some EMs (4/27) described that their partners met friends and drank alcohol after finishing work until late every day. They were afraid that nobody would bring them to hospital for childbirth. Some EMs (6/27) felt stressed and depressed because of their partners’ extramarital sex and were afraid that their partners would have another woman during their pregnancy. Therefore, they often quarrelled with their partners about these matters. Also, some EMs (11/27) felt that their partners did not help to do household work or take care of the child at home.

*We quarrel so often because he always drinks alcohol with his friends in the evening and comes back home at night…I will give birth soon and I am worried that if I will give birth at night, how can I go to the hospital on my own….He was a playboy...I can see his real behaviour.* (EM12, EPDS7)

*My husband drinks alcohol almost every day with his friends and I need to take him back to our home…our relationship is not good because he often drinks alcohol and still contacts his previous partner during my pregnancy.* (EM17, EPDS16)

*My husband wakes up too late, at ten or eleven o’clock. He doesn’t do any housework. I need to wake up early and make breakfast for the children before they go to school.* (EM9, EPDS19)

EMs (14/27) still wanted to help EFs to do work outside during pregnancy. EFs (18/27) felt that the EMs should be more careful when performing household work and outdoor activities to avoid accidents.

*My wife and I mainly stay in the rubber garden; I don’t want her to help me there because she can get abdominal pain. In our garden it is easy to fall, but she still wants to help me. I am very worried.* (EF10)

#### Uncomfortable with in-law family members

Most EMs (19/27) and EFs (19/27) lived in an extended family and had problems in the family. Some EMs (6/27) quarrelled with their in-law family members, such as their parents-in-law or their partners’ relatives when their partners went to work. They often felt uncomfortable because their in-laws were treating them badly. They felt that they could not have a good relationship in the family where they lived. Some EMs (4/27) had thought of or attempted to commit suicide.

*We lived with her [mother-in-law]. She wanted us to leave…She said, “Go out of my land!...She told my husband that I didn’t do housework.* (EM26, EPDS10)

*I once wanted to kill myself during pregnancy…It felt like I had many problems and could not manage them.* (EM11, EPDS7)

### Facing life transition

This category describes life transition during pregnancy. The couples described that having a new child was a transition to new roles and they needed to deal with the situation.

#### Need to prepare for a secure family

During pregnancy, most EMs (20/27) and EFs (20/27) described that they would like to prepare for a secure family. They described a secure family with respect to their own life and their baby. By preparing for a secure family, they would offer a better family to their new child. Some EMs (13/27) reduced the amount of hard work and were more careful in their daily duties. The EMs also made arrangements for their own and their baby’s health. For example, some EMs (6/27) used a private clinic in combination with a government clinic to check their health during pregnancy.

*I visit the antenatal clinic here [community hospital]. I also visit a special private clinic in another district, and will deliver my infant at another hospital. I am sure that I will give birth to a normal child.* (EM2, EPDS8)

The EFs (12/27) felt that thinking about their families and baby’s future, such as planning for the child’s school, would make their family strong. Some EFs (5/27) changed their behaviour even if it was difficult to do so.

*I realise that I drank almost every day. Now I’ve refrained for four days, and during the three-month Buddhism period I intend to refrain from drinking.* (EF12)

#### Dealing with different life events

The EMs experienced both positive and negative events during pregnancy. EMs who graduated from secondary school or higher (13/27) needed ways of dealing with their emotions. Many EMs (18/27) mentioned that increased eating or doing housework was a way of dealing with their situation. They also described that when having unwanted circumstances, such as quarrel with their partners, it could help to take a deep breath, clearing the problem step-by-step and making up their mind to understand the situation. This coping strategy could also solve their emotional problems.

*I don’t want to stay in my house when I am in a bad mood…I usually go to my sister-in-law’s house to shout very loudly, then sit and wait for recovery to get into a good mood.* (EM5, EPDS8)

The EFs (18/27) described that they did not have any problems during their wives’ pregnancy. However, they were concerned about their partners’ problems and encouraged the EMs by giving information, being counsellors, reconciling with the EMs, or buying food for them.

*After she got pregnant, I quarrelled less with her...Nowadays, she is very pleased with me. Close to the date of childbirth, she asked me: “how will we manage”?* (EF25)

#### Accepting changes in life

The EMs and EFs experienced many changes during pregnancy, and they accepted their new and strengthened roles of fully being parents. They tried to see the child’s gender from the first ultrasound scan and if they could not see, they would wait for the next scan. They mentioned that having a boy or girl did not matter because the child would still be their child. The EMs (5/27), especially younger ones, thought about stepping into mother roles, such as taking good care of the child or breastfeeding. First-time EMs (8/27) were looking forward to become a good mother while first-time EFs (6/27) were longing to becoming a family leader. Experienced EMs (11/27) and EFs (9/27) felt that they would be fathers/mothers again.

*I think that even if I was not a good child, I now want to be a good mother…I prefer to breastfeed my child.* (EM20, EPDS8)

*It feels good to be a father soon and I will get a daughter.* (EF4)

### Enhancing confidence

The EMs and EFs described that their trust in persons close to them and in community networks increased during pregnancy. They received verbal encouragement, advice, or material whether they requested it or not.

#### Getting encouragement from surrounding persons

Many EMs (17/27) and EFs (14/27) explained that family members, friends and neighbours supported them during pregnancy, mostly in terms of verbal encouragement and items for the baby. Within couples, the EMs (10/27) and EFs (12/27) described that they accepted each other more. Some EMs (10/27) received help and support from their partners for housework. From outside, the couples were supported by parents, parents-in-law or relatives with, for example, money, materials for the child (e.g. disposable nappies and room), and traditional postpartum care. Friends and neighbours were glad and pleased when the couples were going to have a child, and gave a verbal blessing or celebrated pregnancy.

*If I have problems…I can talk with my neighbours about common topics. I can speak with my brother mainly about money issues, and he supports me financially.* (EM1, EPDS21)

*During pregnancy, I don’t let her cook food. I do it by myself for our family members. I know that pregnant women have difficulty to move. Even walking is difficult for them.* (EF6)

#### Receiving assistance from social networks

Many EMs (15/27) and EFs (14/27) clarified that they got some assistance from health systems, local government or community. They were eligible to receive financial support for hospital care in connection with childbirth, monthly support after childbirth, and loans if needed. EMs (16/27) and EFs (7/27) explained that they received some additional information for maternal and child health by searching on the Internet, including Facebook groups for mothers. Some EMs (13/27) also described that they gained some valuable information from HCPs in primary health centres and hospitals. Bookstores or libraries with free access were also sources of trustworthy information for EFs.

*The hospital promoted me well. I also received ANC services from the health centre. They explained many things to me about child care.* (EM3, EPDS13)

*I get information about how to take care of my wife during pregnancy from my neighbours…We will get financial support from local government after childbirth.* (EF10)

### Dissatisfaction with support

Although there was much support during pregnancy, some EMs and EFs mentioned that they did not receive enough or appropriate support. They wanted to get more services from HCPs and more involvement from their own family and their partner’s family.

#### Needing better healthcare services

Some EMs (8/27) were dissatisfied with the regular ANC services provided by healthcare staff. For example, once when the staff made a mistake about an appointment, the staff did not apologise. Some EFs (9/27) were dissatisfied with the general health service from the hospital. They indicated that some health services should be improved. For example, the staff should be more careful when taking care of mothers and babies. They also would like to get more information related to maternal and child health.

*She [the nurse] saw the calendar and said that the calculation [for the expected date of childbirth] was wrong and she calculated it again. Then she went back and didn’t apologise to me.* (EM8, EPDS13)

*When I went to the hospital, they [the hospital staff] carried my friend’s newborn child without being careful!...Actually, knowing how to take care of a child is important and I should be informed too.* (EF25)

#### Insufficient involvement by the family

Some EFs (3/27) were excluded from their partners’ family because they could not reach the family’s expectations of having permanent job, high education, or high economic status. Therefore, they did not get much support from their partners’ family.

*I am seventeen years old and have graduated from secondary school…I would like to be a member of her family, but her family doesn’t accept me…My father did not feel well when I told him that I will have a child.* (EF4)

## Discussion

The aim of this explorative and descriptive study was to find out life situation and support during pregnancy among EMs with depressive symptoms and their partners. Economic burden for the family was recognised as an obstacle in life for EMs in this study because they had non-permanent occupation and stopped working after pregnancy. Financial constraint has previously been identified as a high stressor for pregnant women [3, 6, 22] and a predictor for depression among women in north-eastern Thailand [6]. Also, living in agricultural settings in Thailand might lead to antenatal depression [15]. In the Thai tradition, a man is always considered as the breadwinner who supports his family members [23]. The EFs in this study who received pay per day expressed that having a child was an economic burden. Economic instability can create or exacerbate parental depression, particularly as couples try to adapt to the growth of family demands after a child’s birth [2].

The EMs in this study shared worries about delivering an abnormal child and having a difficult childbirth. Depressive symptoms have previously been associated with anxiety about neonatal development disorder of the new-born [24] and fear of childbirth [25]. Fear of childbirth can arise, for example, from previous negative obstetric experiences [3]. In this study, the EMs expressed that the fear of a difficult childbirth was based on the information provided by HCPs and other experienced women. The EFs worried mainly about physical risks of the EMs, e.g. haemorrhage. Some studies show that EMs often get worried because of any changes that occur during pregnancy, whereas EFs often think about their family’s future and ways to reduce current problems of EMs [3, 11, 24]. HCPs need to be aware of these worries when providing maternal and child health information to pregnant women and their partners.

In the Thai society, consumption of alcohol is a widely accepted social activity; moreover, Thai people use alcohol as a mode of recreation and way to interact in the community [26]. This behaviour is part of the culture in north-eastern Thailand [27]. It might explain why some EMs in this study were worried about their partner’s daily alcohol consumption after work. Pregnant women have difficult relationships with their partners because of men’s alcohol consumption [15]. Use of alcohol can lead to inappropriate behaviour among men, such as interpersonal violence in the form of verbal abuse when quarrelling, which may influence EMs’ mental well-being [28]. In Thailand, it has been found that many pregnant women (> 60%) drink alcohol and stop when they get pregnant, and more than 50% of pregnant women have low marital satisfaction with their husbands [6]. It may be difficult for the individuals to change drinking behaviour on their own. As this matter is a cause of perinatal depression, it should be taken into consideration by the society [29].

Thai men and women have different views of sexuality. Thai men are widely perceived as having a driving need for sex, whereas Thai women are viewed as having control of their sexual feelings [30]. Some EMs felt stressed and depressed concerning their partners’ extramarital sex, which was a reason for quarrels. In the Thai society, sexuality is linked with the social construction of gender and the expectations among men and women. The habit of extramarital sex among Thai males is common, and it can lead to serious repercussions and family break ups [31]. Talking about sexual behaviour, with focus on both EMs and EFs, might need more attention during ANC in Thailand.

Some EMs experienced conflicts and problems with their in-law families. Having a conflict with the parents-in-law has been associated with antenatal depression in Asian cultures [5], e.g. in Vietnam [28]. It is a cultural norm that women in north-eastern Thailand move to their in-law family after marriage, and after childbirth most women (87%) mainly take care of their children [13]. Suicide is a relatively rare event during the perinatal period [32], but some EMs in this study had thought about and/or attempted to commit suicide. Hence, HCPs should be aware of these culture-related issues in providing care for pregnant women and not only recognize somatic problems [33].

Becoming a parent is a life-changing event. This transition can include great expectations but also increase worry and stress [2, 17]. The EMs in this study experienced both positive and negative life situations during pregnancy, while EFs experienced less problems with life situations. It can be depressing for pregnant women if they are not able to control their life situation during pregnancy and do not get sufficient help to prepare for parenthood [11, 17]. Expectant parents need social, psychological and physical support [15]. In this study, expectant parents received support from many sources, such as friends and their partners. Having a large support network helps to protect against antenatal depression [3] and also against postnatal depression [2].

Most EMs and EFs in this study mentioned that the baby’s gender was not important to them. One explanation for this might be that couples in the north-eastern Thai culture usually have several children in their families, which increases their chances of having both boys and girls. However, in China there is a limitation on how many children one can have [22] and in Vietnam there is a son preference, which is deemed to be a predominant cause of depression [29].

Many EFs in this study were dissatisfied with hospital services and wanted to get more involved in the ANC process in order to receive more information about maternal and child health. Several studies also report that fathers feel like they are invisible when present at an ANC unit, and fathers want to be more involved in the pregnancy process [9, 11]. Hence, HCPs need to become advisors not only to pregnant women but also to the pregnant women’s partners when giving pregnancy-related information [11].

### Strengths and limitations

This study aimed to explore and describe life situation and support among EMs with depressive symptoms and their partners. Use of a qualitative method helped us obtain rich data for understanding of these issues in a north-eastern Thai context. Strengths of this study were that both EMs and EFs participated as informants, and there was a large number of informants. The trustworthiness [34] has been highlighted in several steps: creditability, confirmability, dependability, and transferability.

This study was conducted in north-eastern Thailand, which might limit the transferability to other settings. Further, this study concerned couples. Therefore, the results may not reflect experiences of single EMs with depressive symptoms. Depression in EFs might influence depressive symptoms in EMs, but depression was not assessed for EFs. Even if the EPDS does not rely on somatic symptoms and the choice of cut-off score is well grounded, the EPDS cannot replace a clinical diagnosis. Investigation of life situation and support during pregnancy among EMs and their partners using a questionnaire [13] could provide valuable information in addition to our results.

## Conclusions

The results indicate that EMs with depressive symptoms and EFs might have obstacles in life during pregnancy regarding their economic situation, and fear of health problems and of getting an abnormal child. Further, some EMs worried about their partner’s behaviours, such as their use of alcohol or having extramarital sex. They received support from family and social networks, but some of them were dissatisfied with the support from health care. For instance, many EFs wished to get more health information and to get more involved at the ANC clinics during pregnancy. HCPs should be aware of the influence of culture and contextual factors when providing ANC to pregnant women and their partners. Involvement of EFs in the care must not be neglected.

## Supplementary information

**Additional file 1.** Interview guides for individual interviews with pregnant women and their partners.

## Data Availability

The datasets used and/or analysed during the current study are available from the corresponding author on reasonable request.

## Abbreviations

ANC: Antenatal care
EF: Expectant father
EM: Expectant mother
EPDS: Edinburgh postnatal depression scale
HCP: Healthcare professional
