# Supplementary material for: Life situation and support during pregnancy among Thai expectant mothers with depressive symptoms and their partners: a qualitative study
Source: BMC Pregnancy Childbirth. 2020 Apr 9;20:207. doi: 10.1186/s12884-020-02914-y (PMC7147066; doi:10.1186/s12884-020-02914-y)
Supplement: Supplementary file 1 — Additional file 1. Interview guides for individual interviews with pregnant women and their partners. [file 12884_2020_2914_MOESM1_ESM.docx]

**Interview guides for individual interviews with pregnant women and their partners**

## **Interview guide about life situation and support: Pregnant women**

- Background questions about: Gender, Age, Education, Marital status, Employment, Religion, Number of years (months) of life together, Residential area
- How have you felt during your pregnancy?
- What are your feelings about the new life situation as you soon will get with a newborn baby?
- Which feelings and events have had an effect on your relationships with your husband/partner, children, family, friends or work colleagues? Please give examples.
- Has your mood been going up and down during your pregnancy? If yes, please give examples of situations in which your mood has been going up and/or down.
- What do you think would help (have helped) you cope with your situation?
- What roles do you think expectant parents should have during the pregnancy period? Please describe.
- What kinds of support and service do you need during your pregnancy (husband/partner, family, friends, healthcare, etc.)? Please describe.
- Is there something else that you would like to tell me or that I should know?

## **Interview guide about life situation and support: Partners**

- Background questions about: Gender, Age, Education, Marital status, Employment, Religion, Number of years (months) of life together, Residential area
- How have you felt during your wife’s/partner’s pregnancy?
- What are your feelings about the new life situation you will soon get with a newborn baby?
- Which feelings and events have had an effect on your relationships with your wife/partner, children, family, friends or work colleagues? Please give examples.
- Has your mood been going up and down during your wife’s/partner’s pregnancy? If yes, please give examples of situations in which your mood has been going up and/or down.
- What do you think would help (have helped) you cope with your situation?
- What roles do you think expectant parents should have during the pregnancy period? Please describe.
- What kinds of support and service do you need during your wife’s/partner’s pregnancy (wife/partner, family, friends, healthcare, etc.)? Please describe.
- Is there something else that you would like to tell me or that I should know?
